# Effect of Acute Exercise Mode on Serum Brain-Derived Neurotrophic Factor (BDNF) and Task Switching Performance

**DOI:** 10.3390/jcm7100301

**Published:** 2018-09-24

**Authors:** Chiao-Ling Hung, Jun-Wei Tseng, Hsiao-Han Chao, Tsung-Min Hung, Ho-Seng Wang

**Affiliations:** 1 Department of Athletic, National Taiwan University, Taipei 10617, Taiwan; musehung@ntu.edu.tw (C.-L.H.); nikkichao@ntu.edu.tw (H.-H.C.); 2 Department of Physical Education, National Taiwan Normal University, Taipei 10646, Taiwan; holyspiritfire27@gmail.com

**Keywords:** switch cost, executive function, open-skill, closed-skill

## Abstract

Previous studies have consistently reported a positive effect of acute exercise on cognition, particularly on executive function. However, most studies have focused on aerobic and resistant forms of exercise. The purpose of this study was to compare the effect of ‘open-skill’ with ‘closed-skill’ exercise (defined in terms of the predictability of the performing environment) on brain-derived neurotrophic factor (BDNF) production and task switching performance. Twenty young adult males participated in both closed (running) and open (badminton) skill exercise sessions in a counterbalanced order on separate days. The exercise sessions consisted of 5 min of warm up exercises followed by 30 min of running or badminton. The exercise intensity was set at 60% (±5%) of the heart rate reserve level (HRR) with HR being monitored by a wireless heart rate monitor. Blood samples were taken and participation in a task-switching paradigm occurred before and after each exercise session. Results showed no differences in serum BDNF or task-switching performance at the pre-test stage, however, badminton exercise resulted in significantly higher serum BDNF levels (a proxy for levels of BDNF in the brain) and near significant smaller global switching costs relative to running. This study has provided preliminary evidence in support the relative benefits of open-skills exercises on BDNF and executive function.

## 1. Introduction

Emerging evidence has suggested that exercise may facilitate cognitive performance [1,2]. Within this, there is a growing body of research which looks at how a single bout of exercise affects cognitive performance. In the case of aerobic exercise, the most common exercise mode used in this line of research, it has been shown that 30 min of moderate-vigorous aerobic exercise improves cognitive performance in older adults [3,4], middle-aged adults [5,6], young adults [6,7,8], children [9], and children with attention deficit/hyperactivity disorder [10,11]. Acute resistance exercise has also been shown to be beneficial to cognitive performance [5,12,13].

The type of exercise may be an important factor determining the effects of acute exercise on cognitive performance. Colcombe and Kramer [1] indicated that the effect size of exercise on cognitive function in older adults was larger when combining both cardiovascular and resistance forms of exercise (EF = 0.59) relative to aerobic exercise alone (EF = 0.41). Similarly, Pontifex et al. [14] compared the effect of acute aerobic versus resistance exercise on executive control of working memory, and found that the aerobic exercise-induced changes were more strongly related to executive control.

Beside the comparison between aerobic and resistance exercises, there may also be an important distinction between open and closed skill exercises. The categorization of exercise as being open-skill (e.g., badminton, basketball) or closed-skill (e.g., jogging, golf) is based on the predictability of the performing environment [15]. Huang et al. [16] found that although both open- and closed-skill exercise groups exhibited faster reaction times in the flanker task relative to a group that did not participate in regular exercise, only the open-skill group demonstrated more efficient neural resource allocation. Similarly, using a task-switching paradigm, Dai et al. observed faster reaction times and larger P3 amplitudes in the two exercise groups compared to the irregular exercise group, but only the open-skill group demonstrated additional facilitation effects on global switch costs [17]. The beneficial effects of long-term participation in open-skill exercise were further corroborated by Tsai and Wang [18] in a task-switching paradigm.

Other evidence comes from data on neurochemicals release associated with exercise. Specifically, open skill exercise seems to result in higher levels of exercise-induced brain-derived neurotrophic factor (BDNF) release. BDNF is one of a family of neurotrophic factors that perform diverse functions in brain development and plasticity [19,20] and in humans, levels have been shown to be significantly elevated in response to exercise [21]. Studies involving both acute and chronic exercise found that increased serum concentrations of BDNF were associated with improved medial temporal lobe function and this suggests a possible functional role for this neurotrophic factor in exercise-induced cognitive enhancement in humans [22]. Importantly, previous studies have suggested that enriched environments can enhance the expression of BDNF [23]. Given the difference in the complexity of the environments embedded in open- vs. closed-skill exercises, it is expected that there would be higher levels of BDNF released in the case of the former.

Exercise-elicited cognitive benefits may be disproportionally larger for higher-order cognitive domains such as executive function [24], a meta-level and top-down cognition consisting of multiple mental processes, namely inhibitory control, working memory, and cognitive flexibility, that are involved in goal-oriented behaviors and self-regulation [25]. Task switching paradigms are one of family of tasks that have been extensively used to examine executive function in regard to aspects of working memory, inhibition, and mental flexibility [26]. A typical task switching paradigm consists of three different conditions: Repeated task trials in task-homogenous pure blocks (i.e., AAAA; BBBB), switch trials in task-heterogeneous mixed blocks (e.g., AB or BA), and non-switch or repeated task trials in task-heterogeneous mixed blocks (e.g., AA or BB). Global switch costs refers to reaction time (RT) differences between mixed block and pure block presentations, reflecting the efficiency of maintaining multiple task sets in working memory as well as the selection of the task to be performed next [27]. Local switch costs refer to reaction time differences between non-switching and switching trials under mixed block conditions. These reflect the effectiveness of executive control processes responsible for the activation of the currently relevant task set and the deactivation of the task set that was relevant on the previous trial [27].

Given the limited research on exercise modes other than aerobic or resistance forms, and the possible contribution of BDNF to the relationship between acute exercise and cognitive benefit, this study was aimed at determining the effect of different acute exercise modes (open-skills vs. closed-skills) on serum BDNF levels and task switching performance. It was predicted that participation in acute open-skill exercise would result in greater beneficial effects than participation in closed-skill exercise.

## 2. Method

### 2.1. Participants

Undergraduate and graduate male students were recruited through advertisements. From an original sample of 22 participants recruited, two failed to complete all testing sessions. Only the 20 participants who completed all sessions were included in statistical analysis. To be included in this study, participants had to meet the following criteria: (1) absence of cardiovascular disease, diabetes, history of neurological problems, preexisting injuries, smoking or intake of recreational drugs; (2) right-hand dominant, absence of hearing or vision problems and having normal, or corrected-to-normal, vision, and (3) a score on the Physical Activity Readiness Questionnaire (PAR-Q) indicating that exercise could be performed safely. All participants provided written consent, and the study protocols were approved by the institutional review board of the National Taiwan Normal University (201801HM008). Participants’ demographic characteristics are presented in Table 1.

### 2.2. Acute Exercise Intensity Manipulation Check

In the acute exercise sessions, participants were instructed to engage in 5 min of warm up exercises and 30 min of running or badminton in counterbalanced order [28]. Although the distance travelled while running or engaging in badminton were not measured, both exercises were performed at a moderate intensity. The exercise intensity was based on heart rate reserve (HRR), which was calculated using the formula provided by [29] as ‘Maximal HR’ minus ‘Resting HR’, with ‘Maximal HR’ being estimated by the formula “205.8−0.685 (age)” [30]. The target HR was calculated as follows: Target HR = (HRR × percentage intensity desired) + Resting HR. Moderate intensity was defined as 60% (±5%) HRR.

HR was monitored by a wireless heart rate monitor (BioHarness Team System, Zephyr™, Annapolis, MD, USA). Three HR measures were recorded and these were: HR-rest (HR assessed immediately before conducting the cognitive tasks during the pre-test), HR-exercise (the averaged HR assessed every 30 s during exercise), and HR-post (HR assessed immediately after treatment and before conducting the cognitive tasks at the post-test). The Rating of Perceived Exertion (RPE) scale, which ranges from 6 to 20, was used to provide a subjective rating of each individual’s perception of effort during the exercise [31]. RPE was recorded both before and during exercise.

### 2.3. Blood Sampling and Analysis

During each exercise session, venous blood samples were obtained immediately before and after exercise. Blood was drawn from the antecubital vein of each participant’s non-dominant hand and collected in 4 mL green vacutainers and 6 mL red vacutainers. The blood in the green vacutainers, used to analyze the hematocrit level, was separated using a centrifuge at 4000 r/min for 10 min. The blood in the red vacutainers was first stored at 4 °C in a refrigerator for 60 min in order to induce clotting and separated using a centrifuge at 4000 r/min and 4 °C for 10 min. Supernatant fluid was then extracted and stored at −70 °C. An enzyme-linked immunoassay (ELISA) was conducted, using a BDNF Sandwich ELISA Kit (Millipore, Burlington, VT, USA) as the reagent to analyze BDNF levels. Participants were instructed to avoid strenuous exercise for 24 h prior to the study exercise session, while food, caffeine, and alcohol intake were also prohibited for 8 h prior to each session.

### 2.4. Cognitive Task

Cognitive performance was assessed using the task switching paradigm, modified by Hung et al. [11], and controlled via Neuroscan Stim software (version 2.0; Neuro Inc., El Paso, TX, USA). The task including pure and mixed task conditions in three subtests. In each task, a white numeric digit (digits 1–9, excluding 5) was presented in the center of a computer screen on a black background. Pure task conditions included two subtests. For the first subtest, designated as being of the ‘A’ type, participants had to identify whether the number surrounded by a solid-line rectangle, was smaller (1, 2, 3, 4) or larger (6, 7, 8, 9) than 5 (i.e., all tasks were of the ‘A’ type). For the second subtest, designated as being of the ‘B’ type, the participants had to identify whether the number, surrounded by a dashed square, was odd (1, 3, 7, 9) or even (2, 4, 6, 8) (i.e., all tasks were of the ‘B’ type). For the mixed-task condition, the two previous tasks were combined with an alternative run switching paradigm (i.e., AABBAA…) [32]. The participants were instructed to use their index fingers of each hand to, as quickly and accurately as possible, press either the ‘X’ key to indicate smaller or odd, and the ‘M’ key to indicate larger or even, in the ‘A’ or ‘B’ tasks respectively. The probability of a correct response requiring action by the right or left hand was the same.

Each of the 8 digits within each task appeared with equal probability, in random order. The digits were presented for 200 ms, with a 2000 ms response-stimulus interval. If no response was made, the trial was terminated 3000 ms after the onset of stimuli. Participants completed 64 trials in each of the pure task conditions and 128 trials (64 trials × 2 blocks) in the mixed-task condition. The first trial in each block for both pure task and mixed-task conditions was discarded from the analyses. The viewing distance was approximately 70 cm and visual angles were 4.6°. Measures were taken of reaction time (RT), and response accuracy in pure, mixed, non-switching and switching trials. Global switch cost was calculated as the difference in RT between the pure and mixed blocks (i.e., the averaged first and second blocks). Local switch cost was calculated as the difference in RT between the non-switch and switch trials in the mixed blocks.

### 2.5. Procedure

All participants were instructed by the experimenter to complete several questionnaires to ensure that they met the specified inclusion criteria. After screening, participants individually attended the laboratory for two testing sessions with a 7-day interval between sessions. The order of running and badminton exercise sessions was counterbalanced across participants. Additionally, all testing was performed at the same time of the morning to control for circadian influences. Three stages were involved in the experimental procedure: (1) pre-test, (2) running/badminton session, and (3) post-test.

At the pre-test stage, each participant sat resting in a sound proof room wearing a HR monitor. A 10 mL blood sample was taken and then the resting HR was determined after 5 min of monitoring. The participant then engaged in the pre-test stage of the cognitive test.

Participants were then instructed to perform a single bout of aerobic exercise, either badminton or running. In the running session, participants were instructed to run on an indoor track, while in the badminton session, participants were instructed to hit the ball served by an experienced badminton coach. The exercise protocol included 5 min of warm up, 30 min of the main exercise, and then a 5 min cool down period. Heart rate measures were taken during the exercise period.

After the exercise session was completed, another 10 mL blood sample was drawn from each participant who was then taken back to the pre-test room and allowed to rest until their HRs returned to within 10% of their pre-exercise levels [33]. Participants were then asked to perform the same cognitive tasks administered during the pre-test stage.

### 2.6. Statistical Analysis

To test the exercise intensity manipulation between the running and badminton session, a paired *t*-test was applied to compare HR-exercise. Pre and post exercise levels of serum BDNF and RPE were analyzed with a 2 (Exercise sessions: badminton, running) × 2 (time: pre, post) two-way repeated-measure ANOVA.

For cognitive task performance, separate analyses were conducted to examine global and local RT and accuracy switch effects. First, a paired *t*-test was used for testing task performance during the pre-test phase. If there were no differences at the pre-test stage, two post-test repeated measures ANOVAs were conducted, global switch effect was determined by a 2 (Exercise session: badminton, running) × 2 (Condition: pure and mixed trials) analysis while the local switch effect was examined by a 2 (Exercise session: badminton, running) × 2 (Condition: non-switching and switching trials in the mixed condition) analysis. If a significant pre-test difference was detected, then it was intended that the variable would be used as a co-variate in a two-way ANOVA. In addition, to compare switch costs, separate paired *t*-tests were used to analyze global (the difference in RT between mixed and pure conditions) and local (the difference in RT between non-switching and switching trials in the mixed condition) switch costs between the exercise types (badminton vs. running). For significant interactions and main effects, multiple comparisons were performed using Bonferroni post-hoc analysis. An alpha of 0.05 was used as the level of statistical significance for all analyses.

## 3. Results

### 3.1. Exercise Intensity Manipulation

Paired *t*-tests found no difference for HR-exercise (*t* (19) = −0.86, *p* > 0.05) between badminton (M = 140.58 bpm, S.E. = 2.22) and running (M = 141.80 bpm, S.E. = 1.38). Results also showed no difference for the time interval (*t* (19) = −0.28, *p* > 0.05) needed to return to within 10% of pre-exercise HRs between badminton (M = 15.21 min, S.E. = 2.00) and running (M = 15.95 min, S.E. = 1.69). For RPE, a 2 × 2 two-way repeated-measure ANOVA revealed that there was no significant main effect of type (*F* (1,19) = 0.00, *p* > 0.05, $\eta_{p}^{2}$ = 0.00) or interaction (*F* (1,19) = 0.74, *p* > 0.05, $\eta_{p}^{2}$ = 0.04). These results indicate that the two activities involved similar levels of exercise intensity. The main effect for time was significant (*F* (1,19) = 88.43, *p* < 0.001, $\eta_{p}^{2}$ = 0.82), with higher RPE being observed in during exercise (M = 12.70, S.E. = 0.55) compared with pre-test (M = 8.93, S.E. = 0.42).

### 3.2. BDNF Response

**Figure 1:**
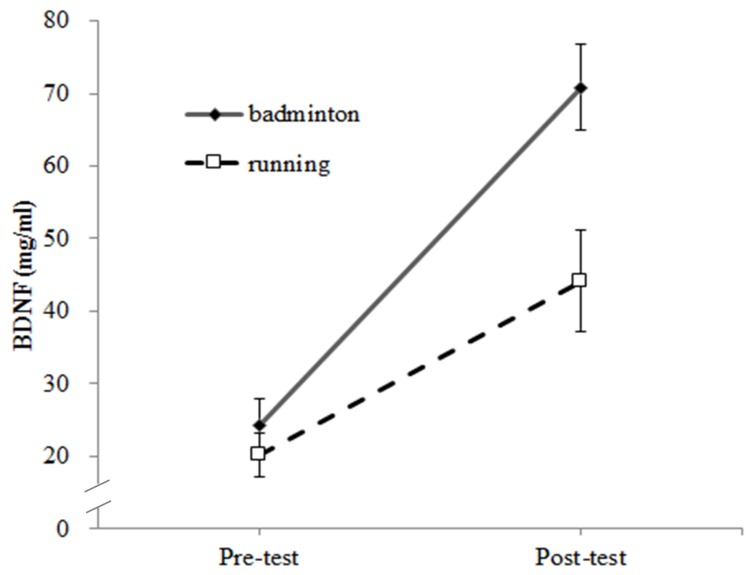
Effects of different acute exercise sessions on serum BDNF in pre and post exercise.

Serum BDNF showed a significant interaction of exercise session by time (*F* (1,19) = 9.51, *p* < 0.01, $\eta_{p}^{2}$ = 0.33), a follow-up simple main effects analysis was used to disaggregate this interaction effect, and a significant time effect was found for the badminton (*t* (19) = −6.45, *p* < 0.001) and running (*t* (19) = −4.06, *p* < 0.001), with higher serum BDNF at post-test than pre-test. In addition, while there were no significant pre-test differences in serum BDNF, the badminton group had higher post-test serum levels (*t* (19) = 3.53, *p* < 0.01) (Figure 1).

### 3.3. Behavioral Data

The paired *t*-test data found no pre-test differences between exercise types (*ts* (19) = −1.09 ~ 1.4, *p* > 0.05) in RT and accuracy. Furthermore, no significant differences in global and local costs (*ts* (19) = −0.78 ~ −0.93, *p* > 0.05) were found (Table 2). Therefore, a 2 × 2 repeated measures ANOVA was applied to the post-test data. Table 3 presents the detailed behavioral data for the task switching parameter for post-exercise session.

#### 3.3.1. Global Switch Effect

The RT analysis revealed neither interactions (*F* (1,19) = 4.31, *p* > 0.05, $\eta_{p}^{2}$ = 0.18) nor exercise session (*F* (1,19) = 0.11, *p* > 0.05, $\eta_{p}^{2}$ = 0.01) effects. For response accuracy there was a similar lack of evidence of interaction (*F* (1,19) = 0.63, *p* > 0.05, $\eta_{p}^{2}$ = 0.03) or main effects of exercise session (*F* (1,19) = 1.83, *p* > 0.05, $\eta_{p}^{2}$ = 0.09).

#### 3.3.2. Local Switch Effect

The RT analysis found no interactions (*F* (1,19) = 0.08, *p* > 0.05, $\eta_{p}^{2}$ = 0.004) or main effects for exercise session (*F* (1,19) = 1.15, *p* > 0.05, $\eta_{p}^{2}$ = 0.06) with a similar pattern found in the case of response accuracy interactions results (*F* (1,19) = 2.13, *p* > 0.05, $\eta_{p}^{2}$ = 0.10) and main effect results (*F* (1,19) = 1.64, *p* > 0.05, $\eta_{p}^{2}$ = 0.08).

#### 3.3.3. Switch Cost Effect

Regarding global switch costs, paired *t*-test found a near-significant difference (*t* (19) = −2.08, *p* = 0.052) between the two exercise sessions. Examination of the means indicates a smaller global switch cost following badminton (M = 178.93 ms, S.E. = 14.83) relative to running (M = 214.81 ms, S.E. = 14.83). In contrast, no difference in local switch cost was observed (*t* (19) = 0.28, *p* > 0.05).

## 4. Discussion

The main objective of the present study was to compare the effect of acute open-skill exercise with closed skill exercise on BDNF and task switching performance. Findings indicated that acute badminton exercise has resulted in significantly higher serum BDNF and near significant smaller global switching costs compared with running.

Serum BDNF level increased from baseline in both exercise types, indicating that an acute bout of exercise increased serum BDNF concentrations. This finding is consistent with past studies that showed serum BDNF levels in humans were significantly elevated in response to an acute bout of exercise [21,22]. Importantly, exercise mode moderated the effect of exercise on BDNF release with participation in badminton resulting in significantly higher serum BDNF concentrations than running. This finding is in line with previous animal studies that suggested that an enriched environment can enhance the expression of BDNF [23]. In that particular study, the animals were living in a highly varied and interesting cage environment (e.g., tubes, ropes, and obstacles) that promoted enriched sensory, cognitive, and motor experiences. Furthermore, the effects of physical activity and environmental enrichment are additive for the integration and survival of newly generated neurons [34]. Although serum BDNF levels are only a proxy for the levels of BDNF in the brain, our study showed that serum BDNF is sensitive to the effect of exercise mode and 30 min of open-skills exercise stimulate relatively more BDNF production. This finding implies that exercise mode might be a moderating factor for the relationship between acute exercise and BDNF level. 

With regard to global and local switching effect, no differences were observed between running and badminton sessions. This result is consistent with the findings of previous studies which examined the effect of acute exercise and executive control in young adults using a task switching paradigm [35]. Different acute exercise sessions do not appear to exert differential effects on global and local switching performance, indicating that these two indices were not sensitive to the different acute exercise sessions. One possibility is that this was due to the fact that participants were cognitively healthy young adults (mean age = 23.1), already functioning near the peak of cognitive ability and therefore with little room for improvement [36].

Although not reaching significance, the observed tendency towards lower global switch costs after badminton is nevertheless worthy of discussion. Our results are consistent with previous studies using a task switching paradigm examining the relationship between physical activity and cognition, which found that global switch costs are more sensitive to exercise [17,37,38]. The finding also corroborates previous research showing that older adults engaging in cyber cycling, an exercise with more cognitive loading, achieved better cognitive function than traditional exercisers, for the same effort [39]. Global switch costs are an index of efficiency in maintaining multiple task sets in working memory as well as an index of the efficiency of executive processing [27,40]. Accordingly, the beneficial effects of open-skill exercises on global switch costs could stem from an association between working memory and exercise type. Previous meta-analyses found that athletes engaging in interceptive sports (e.g., tennis, badminton, fencing) had larger effects than participants in strategic (e.g., volleyball and basketball) or static sports (e.g., long distance running, swimming) on attentional cuing, processing speed, and other attention paradigms of cognitive tasks [41].

The finding of no differences in local switch costs between the two exercise sessions was expected since this has been reported by previous cross-sectional studies [17,38,42]. Specifically, Dai et al. [17] found greater effects on global switch costs from open-skill exercises, but no differences in local switch costs were found among open-skill, closed-skill, and irregular-exercise groups. Local switch costs refer to the timing of a task-set reconfiguration process and are measured as the difference between switch and non-switch trials within the same mixed block [32,43]. It was proposed that switch costs reflect a measure of cognitive rigidity (difficulty in abandoning the currently irrelevant task causing proactive interference) [43] or flexibility (time-consuming process of reconfiguration of task sets) [32]. The present study suggests that while all forms of acute exercise influence executive control, different type of exercise may result in different effects on executive function as measured by task-switching costs.

Although the present study suggests links among acute exercise mode, serum BDNF, and the switching subcomponent of executive function, there are some limitations to the interpretation of these results. First, there are other biomarkers affected by acute exercise, such as insulin-like growth factor-1 (IGF-1) that provide trophic support to the brain [44]. Serum IGF-1 levels are positively correlated with cognitive performance in healthy older people [45]. The current study was also limited by its relatively small sample size. In addition, the results of the current study cannot be generalized to females. The decision to only recruit males was based on the results of past studies which reported that BDNF levels vary during the different phases of the menstrual cycle, with those with premenstrual syndrome (PMS) showing a different pattern relative to women without PMS [46]. Moreover, the present study compared only the two exercise modes without a control condition. It is suggested that future studies include a no-exercise control condition to examine the magnitude of change from acute exercise. Finally, there may be both sport-specific and more general cognitive enhancements resulting from competitive sport training. It would be interesting to examine the relationship between sport expertise and measures of cognition that presumably tap into some of the fundamental cognitive demands of competitive sport training [47]. Exercise intervention has been shown to enhance selective attention/conflict resolution, and associative memory in seniors with MCI (mild cognitive impairment) [48]. As such it would also be of interest to try to replicate the current study’s results with an elderly population and/or those experiencing reduced executive functioning, such as MCI and dementia.

## 5. Conclusions

In conclusion, this is the first study that has examined the differential benefit between open and closed skill modes of acute exercise on executive function combining biochemical indicators and behavioral outcomes. Results provide preliminary support for a moderating role of type of exercise on the relationship between acute exercise and cognitive function. Our data corroborate the results of a previous cross-sectional study [17], in finding that acute open-skill exercise produces a relatively greater level of enhancement in BDNF level, and may also differentially facilitate task switching performance. These results may have important public health implications in suggesting that relatively greater exercise related cognitive benefits may result from participation in open-skill activities. Future studies on the long-term effects of open and closed-skill exercises, especially for older adults, are recommended.

## Figures and Tables

**Table 1:** Participants’ demographic characteristics.

Variable	Total
Sample size	20
Age (years)	23.15 ± 2.48
Height (m)	1.75 ± 0.07
Weight (kg)	68.98 ± 8.86
BMI (kg/m^2^)	22.49 ± 2.10
HR-rest (bpm)	60.75 ± 8.94
HR-max (bpm)	189.95 ± 1.67
60% HRR (bpm)	138.30 ± 3.64

BMI = body mass index; bpm = beats per minute; HR = heart rate; HR-rest = HR assessed immediately before conducting the cognitive tasks at the pre-test; HR-max = the averaged maximal HR; HRR = the averaged heart rate reserve at 60%.

**Table 2:** Reaction time and accuracy for global switch and local switch among exercise session in pre-test (mean value ± standard deviation).

Variable	Pre-Test			
	Open-Skill	Closed-Skill	*t*	*p*
Global switch RT (ms)				
Pure	475.625 ± 56.02	485.12 ± 81.07	−0.66	0.52
Mixed	727.13 ± 151.03	766.21 ± 151.61	−0.98	0.34
Global switch cost	251.51 ± 123.6	281.09 ± 108.37		
Local switch RT (ms)				
Non-switch	477.54 ± 78.51	740.3 ± 142.89	−0.86	0.4
Switch	656.47 ± 124.58	792.12 ± 164.94	−1.09	0.29
Local switch cost	41.23 ± 65.46	51.83 ± 57.46		
Global switch accuracy (%)				
Pure	96.2 ± 3.17	93.35 ± 11.42	1.09	0.29
Mixed	92.3 ± 5.54	89.02 ± 11.43	1.26	0.22
Local switch accuracy (%)				
Non-switch	93.2 ± 6.72	90.85 ± 12.08	0.94	0.36
Switch	91.5 ± 5.66	87.85 ± 11.05	1.4	0.18

**Table 3:** Summary of analyses from two-way ANOVA (Exercise session × Condition) in post-test (mean value ± standard deviation).

Variable	Post-Test						Sessions *p*-Value	Interactions *p*-Value
	Conditions	Open-Skill	Closed-Skill	Conditions	Open-Skill	Closed-Skill		
Global switch RT (ms)	Pure	477.54 ± 78.51	485.12 ± 81.07	Mixed	656.47 ± 124.58	766.21 ± 151.61	0.74	0.05
Local switch RT (ms)	Non-switch	639.64 ± 115.31	663.69 ± 120.1	Switch	673.29 ± 136.77	694.49 ± 120.54	0.3	0.78
Global switch accuracy (%)	Pure	95.55 ± 3.83	96.45 ± 3.24	Mixed	91.5 ± 7.21	93.35 ± 3.69	0.19	0.44
Local switch accuracy (%)	Non-switch	94.5 ± 4.25	94.45 ± 4.21	Switch	88.5 ± 13.18	92.35± 3.91	0.22	0.16
